# Supplementary material for: Velocity bias in intrusive gas-liquid flow measurements
Source: Nat Commun. 2021 Jul 5;12:4123. doi: 10.1038/s41467-021-24231-4 (PMC8257743; doi:10.1038/s41467-021-24231-4)
Supplement: Supplementary file 1 — Supplementary Information [file 41467_2021_24231_MOESM1_ESM.pdf]

Supplementary Information: Velocity bias in intrusive gas-liquid flow measurements

B. Hohermuth[a*], M. Kramer[b], S. Felder[c], D. Valero[d]

[a*] *Laboratory of Hydraulics, Hydrology and Glaciology (VAW), ETH Zurich, Zurich, Switzerland, hohermuth@vaw.baug.ethz.ch, corresponding author*
[b] *School of Engineering and Information Technology (SEIT), UNSW Canberra, Campbell, Australia, m.kramer@adfa.edu.au*
[c] *Water Research Laboratory, School of Civil and Environmental Engineering, UNSW Sydney, Sydney, Australia, s.felder@unsw.edu.au*
[d] *Water Resources and Ecosystems Department, IHE Delft Institute for Water Education, Delft, the Netherlands, d.valero@un-ihe.org*

Supplementary Figures

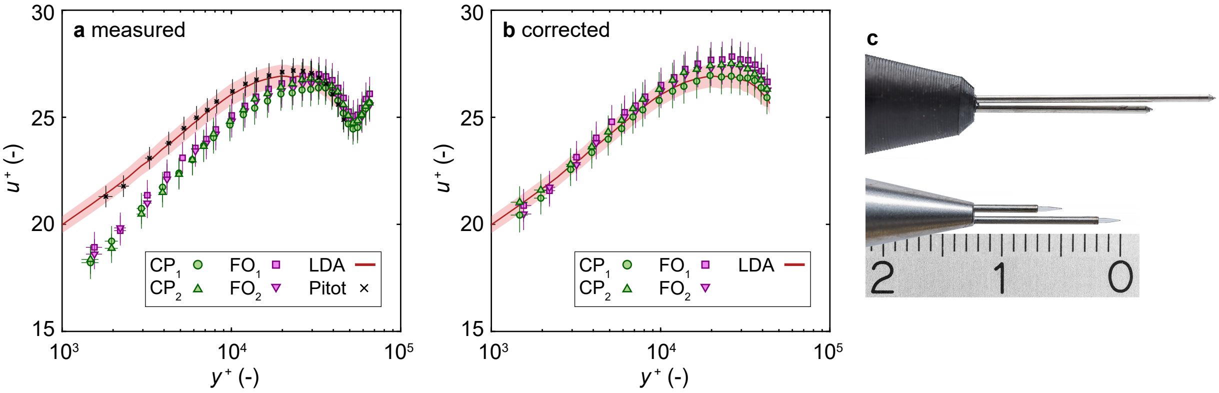

Supplementary Figure 1: Comparison of continuous- and dispersed-phase velocities for Re $= 1.3 \cdot 10^6$. The continuous-phase velocities were measured with LDA and a Pitot tube, whereas the dispersed-phase velocities were obtained with two conductivity phase-detection intrusive probes (CP_1, CP_2) and two fibre-optical phase-detection intrusive probes (FO_1, FO_2). The shear velocity (u^*) obtained by LDA was used to scale all measurements as $u^+ = U/u^*$, $y^+ = y u^*/\nu_c$, with U = mean velocity and ν_c = kinematic continuous phase (water) viscosity. **a** The measured dispersed-phase velocities showed consistent results for all four phase-detection intrusive probes with FO probes yielding slightly larger velocities than CP. However, the dispersed-phase velocities were underestimated compared to the LDA and Pitot tube data. The excellent agreement of LDA and Pitot tube continuous-phase velocities confirms that the LDA acquisition parameters were appropriate for bubbly flow conditions. Error bars indicate standard error; for LDA data, the standard error is shown as shaded area. **b** The corrected dispersed-phase velocities were in good agreement with the continuous-phase velocities, confirming the suitability of the correction scheme for different probe types. In the near-wall region ($y^+ \lesssim 5 \cdot 10^3$), corrected velocities measured by FO probes were slightly smaller than CP values due to the difficulty of selecting an appropriate representative outer diameter Φ_o for the FO probe. Error bars indicate standard error; for LDA data, the standard error is shown as shaded area. **c** Photos of CP_1 and FO_1; in contrast to the constant Φ_o for CP probes, the outer probe diameter of FO probes is not constant. Scale bar ticks correspond to 1 mm.

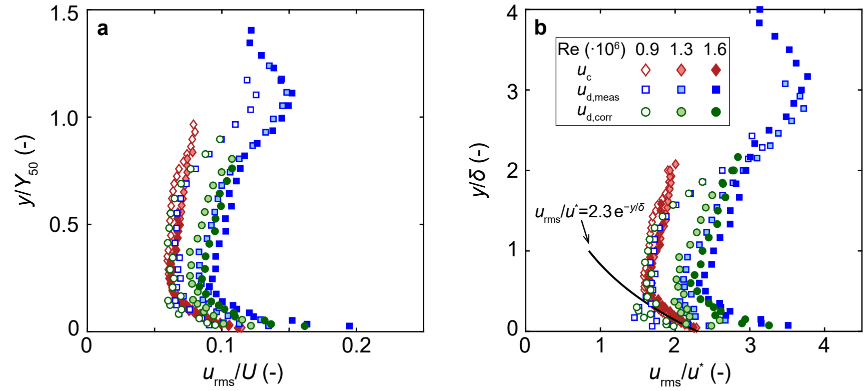

Supplementary Figure 2: Turbulence intensity of the continuous-phase velocity (u_c) recorded with LDA and the dispersed-phase velocity measured with a conductivity phase-detection intrusive probe (CP_1) with ($u_{d,corr}$) and without ($u_{d,corr}$) bubble-probe interaction correction. The velocity standard deviation (u_{rms}) for the continuous phase was directly obtained from the measured LDA velocity time series using residence time weighting [1]. For the dispersed-phase, u_{rms} values were extrapolated to single particles using the AWCC [2]. In **a**, u_{rms} was scaled with the mean (time-averaged) velocity (U) and in **b** with the shear velocity (u^*) estimated from LDA data using the Clauser method; Y_{50} is the location where $C = 0.5$, δ is the the boundary layer thickness set to the location where $U = U_{max}$ to account for velocity-dip effects [3]. The standard errors in **a** and **b** are comparable to the symbol size and thus not shown. The continuous-phase turbulence intensity was similar for all tested flow conditions and compared well with single-phase estimations up to $y/\delta < 0.4$ (solid line in **b** [4]). The almost constant value attained for $y/\delta \geq 0.4$ was likely due to 3D effects caused by the small aspect ratio of the chute. The uncorrected dispersed-phase turbulence intensity was larger than continuous-phase values and increased with increasing Reynolds numbers, corresponding to higher void fractions. Applying the correction scheme reduced the dispersed-phase turbulence intensities, however, differences in velocity fluctuations between the continuous and the dispersed phases were still present. These differences may be caused by (i) instrumentation and 3D flow effects and/or (ii) turbulence modulation. At the current stage, we cannot differentiate between these mechanisms due to limitations in the design of phase-detection intrusive probes.

Supplementary Notes

Supplementary Note 1: Interface detection using cross-correlation analysis

When a bubble is pierced by a double-tip phase-detection probe, changes in the leading and trailing tip voltage signal allow to infer a velocity for each interface. Ideally, the travel times of the first and the second bubble interface ($\mathcal{T}_1$ and $\mathcal{T}_2$) are identical, while bubble deformation and deceleration, signal noise, etc., may lead to differences.

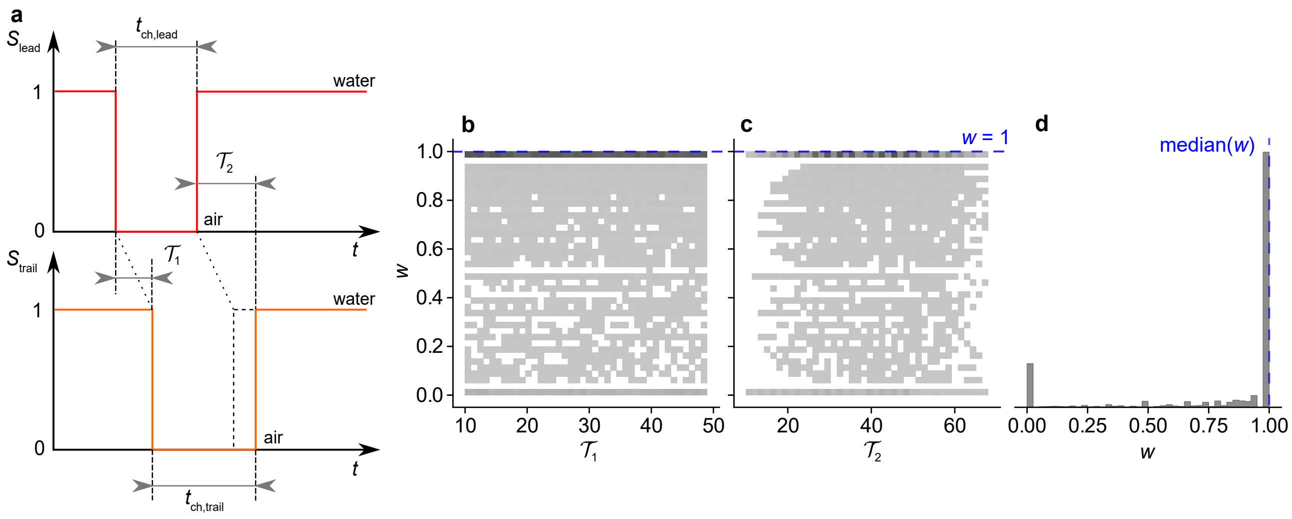

Supplementary Figure 3: Results of the Monte-Carlo simulations for interface detection: **a** variables definition, **b** bivariate histogram ($\mathcal{T}_1$ and w), **c** bivariate histogram ($\mathcal{T}_2$ and w), and **d** univariate histogram ($\mathcal{T}_1$ and w). Darker colors indicate higher frequency in the bivariate histograms, parameters defined in the text.

To estimate bubble velocities, the phase-detection probe signals (phase functions) are broken into small windows, each containing a small number of particles [5; 2]. These window subsignals (S_{lead} and S_{trail}, for leading and trailing tip subsets, Supplementary Fig. 3a) are analysed using a cross-correlation technique, which provides a smooth function displaced by a certain time lag (i.e., correlation coefficient vs lag time). The lag time with maximum correlation coefficient indicates the most probable travel time of the bubble ($\mathcal{T}_{\text{meas}}$). Several factors may affect the location of the correlation peak, for example the deformation of a bubble can elongate the particle signature in S_{trail} (hence $\mathcal{T}_2 \neq \mathcal{T}_1$, Fig. 3a), or electrical noise in S_{lead} and S_{trail} may lead to arbitrary peaks in the cross-correlation function. For the developed correction scheme, it is important to determine which travel time (first or second bubble interface) is detected by the cross-correlation analysis, as specified through the weighting factor w:

$$\begin{aligned}\mathcal{T}_{\text{meas}} &= w\,\frac{2\Delta x}{u_{\text{d,corr}} + u_{\text{d,1,trail}}} + (1-w)\,\frac{2\Delta x}{u_{\text{d,2,lead}} + u_{\text{d,2,trail}}} \\ &= w\mathcal{T}_1 + (1-w)\mathcal{T}_2\end{aligned} \tag{1}$$

Herein, we performed a Monte Carlo simulation with 20,000 samples. Each simulation included a pair of synthetic signals (S_{lead} and S_{trail}) and each synthetic signal contained one bubble event (phase function change from 1 to 0). The basic parameters, as presented in Supplementary Fig. 3a, were simulated as follows: the time distance from origin to the first interface uniformly ranged between 10 to 50 time units (t.u.); the travel time of the first interface $\mathcal{T}_1$ uniformly ranged between 10 to 50 t.u.; the particle chord time of the leading tip $t_{\text{ch,lead}}$ uniformly ranged between 10 to 50 t.u.; the particle chord time of the trailing tip $t_{\text{ch,trail}}$ uniformly ranged between $t_{\text{ch,lead}}$ to $t_{\text{ch,lead}}$+20 t.u.; the travel time of the second interface $\mathcal{T}_2$ was calculated as: $\mathcal{T}_2 = \mathcal{T}_1 + t_{\text{ch,trail}} - t_{\text{ch,lead}}$. In addition, a Gaussian uncorrelated noise was superimposed to the phase functions, having a maximum amplitude between 0 and 5 % of the signal's amplitude. For each simulation, the weighting factor w was evaluated through a comparison of the numerical solution of Supplementary Eq. (1) with the measured travel time $\mathcal{T}_{\text{meas}}$, obtained via cross-correlation analysis. The results of the Monte Carlo simulation (Supplementary Fig. 3d) indicated that $w \approx 1$ on median terms – irrespective of the values of $\mathcal{T}_1$ or $\mathcal{T}_2$ (Supplementary Fig. 3b, c) – implying that the first particle interface defined the cross-correlation output, i.e., $\mathcal{T}_{\text{meas}} \approx \mathcal{T}_1$.

Supplementary Note 2: Bubble column data and temporal evolution of bubble velocity

To assess the suitability of our simplified force balance (Eq. 3) to a wider range of flow conditions, we reanalysed bubble column data from Vejrazka et al. [6]. While our data set covered horizontal flows with medium to high Reynolds numbers with negligible phase-slip, the data of Vejrazka et al. [6] extended the flow conditions to buoyancy-driven vertical flows with small Reynolds numbers and high phase-slip. The data set of Vejrazka et al. [6] comprised detailed high-speed videos of bubbles impinging the tip of a single tip fibre-optical phase-detection intrusive probe. For several bubble diameters, the bubble velocity during the interaction $u_d(t)$ was calculated considering a drag coefficient C_d after Moore [7], a bubble aspect ratio E after Aoyama et al. [8], a virtual mass coefficient C_{vm} after Lamb [9] and the deformation coefficient after Supplementary Eq. (15) with $C_\sigma \approx 0$. Supplementary Figures 4a, b illustrate the result of the comparison of the data of Vejrazka et al. [6] with Eq. (3), comprising the comparison of velocities (Supplementary Fig. 4a) and bubble deceleration (Supplementary Fig. 4b). The comparison of relative velocities $u_{d,2,lead}/u_{d,corr}$ showed close agreement for small bubble diameters, while Eq. (3) slightly underestimated the velocities for larger diameters (Fig. 4a). The small differences were likely caused by inaccuracies in the semi-empirical coefficients since C_d is underestimated for large bubbles, while C_{vm} is overestimated (see Supplementary Figs. 7a, b). Note that Verjrazka et al. [6] developed an analytical force balance expression to obtain $u_{d,2,lead}$ by setting $F_\sigma = F_{vm}$ and calculating E after Moore [7]. Their results differed slightly from ours because they defined the bubble-probe interaction time based on $u_{d,corr}$ while we defined it with $(u_{d,corr} + u_{d,2,lead})/2$ for single tip probes. The evolution of the bubble velocity measured by high-speed imaging in Verjrazka et al. [6] showed an almost linear deceleration (Supplementary Fig. 4b) for different tested bubble diameters. Overall, a good agreement with the numerical solution of Eq. (3) was observed.

The deceleration of bubbles along the probe tips of a phase-detection probe was also calculated with Eq. (3) for flow conditions similar to our experiments, i.e., slightly contaminated water and high velocity. The results of this analysis showed an almost linear deceleration irrespective of the bubble sizes (Supplementary Fig. 4c). The combined analysis of the bubble column data of Verjrazka et al. [6] and our high-velocity data suggest that the deceleration process along phase-detection probes is linear.

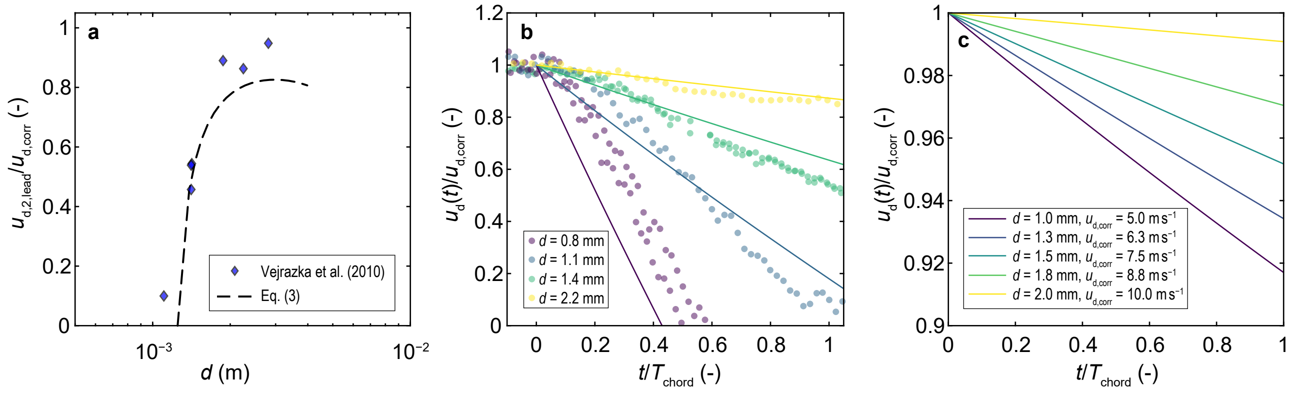

Supplementary Figure 4: Comparison of numerical solution of force balance (Eq. 3) with bubble column data of Vejrazka et al. [6]. **a** ratio of bubble velocity at the end of interaction and before interaction versus bubble diameter. **b** temporal evolution of measured bubble velocities (Vejrazka et al. [6], dots) compared with numerical solution of Eq. (3) (solid lines). T_{chord} denotes chord time, i.e., the time from piercing of the first to piercing of the second interface by the leading tip. **c** temporal evolution of bubble velocities for high-velocity and high Re_b bubble-probe interaction obtained from numerical solution of Eq. (3) with C_σ after Supplementary Eq. (15), C_d after Supplementary Eq. (7), C_{vm} = 0.5 and tap-water conditions.

Supplementary Note 3: Bubble force balance

In this section, we derive the bubble force balance and justify the simplifications embraced in the correction scheme. The preferred modelling strategy for dispersed turbulent flows is the Lagrangian point-particle approach [10], which is applicable to bubbles with a time scale considerably larger than the Kolmogorov time scale. This approach requires an equation of motion for bubbles of diameter d, coupled with the continuous-phase. Herein, we consider a bubble being pierced by the needles of a phase-detection probe and apply Newton's second law to a control volume around a spherical bubble of mass M:

$$M\frac{\mathrm{d}\mathbf{u}_\mathrm{d}}{\mathrm{d}t} = \sum \mathbf{F} \tag{2}$$

where $\mathbf{u}_\mathrm{d}$ is the velocity vector of the bubble relative to an inertial reference frame and $\mathbf{F}$ the vector of body and surface forces acting on the control volume. The mass in the control volume is: $M = V\rho_\mathrm{d}$, with $V = \pi d^3/6$ and ρ_d the dispersed-phase density. Relevant forces comprise (i) forces of bubbles freely transported by the water flow [10; 11] and (ii) forces due to bubble-probe interaction [6; 12]:

$$\sum \mathbf{F} = \mathbf{F}_\mathrm{g} + \mathbf{F}_\mathrm{p} + \mathbf{F}_\mathrm{grad} + \mathbf{F}_\mathrm{d} + \mathbf{F}_\mathrm{vm} + \mathbf{F}_\mathrm{B} + \mathbf{F}_\mathrm{wall} + \mathbf{F}_\sigma + \mathbf{F}_\mathrm{stag} + \mathbf{F}_\mathrm{surf} \tag{3}$$

where $\mathbf{F}_\mathrm{g}$ is the body force due to gravity, $\mathbf{F}_\mathrm{p} + \mathbf{F}_\mathrm{grad}$ are the forces due to pressure, local and convective acceleration in the undisturbed continuous phase, $\mathbf{F}_\mathrm{d}$ is the steady state drag, $\mathbf{F}_\mathrm{vm}$ is the virtual mass, $\mathbf{F}_\mathrm{B}$ is the Basset force, and $\mathbf{F}_\mathrm{wall}$ are the wall forces (including lift and lubrication). We assume $\mathbf{F}_\mathrm{wall} \approx 0$ at distances larger than 1 to 2 d [13]. Next to the wall, $\mathbf{F}_\mathrm{wall}$ results predominantly in bubble movement normal to the probe-wise direction, leading to single tip impacts, which are anticipated to be filtered by the implemented AWCC technique. The bubble-probe interaction comprises surface tension forces $\mathbf{F}_\sigma$, stagnation pressure forces $\mathbf{F}_\mathrm{stag}$ and surface deformation forces $\mathbf{F}_\mathrm{surf}$. All force terms in Supplementary Eq. (3) are detailed in the following. Using Archimedes' principle, the body and pressure forces are expressed as:

$$\mathbf{F}_\mathrm{g} + \mathbf{F}_\mathrm{p} = -\frac{\pi d^3}{6}(\rho_\mathrm{c} - \rho_\mathrm{d})\,\mathbf{g} \tag{4}$$

being ρ_c the continuous-phase (water) density and $\mathbf{g}$ is the gravity vector. The undisturbed flow velocity gradients acting over the control volume affect the bubble dynamics through [10]:

$$\mathbf{F}_\mathrm{grad} = \frac{\pi d^3}{6}\rho_\mathrm{c}\frac{\mathrm{D}\mathbf{u}_\mathrm{c}}{\mathrm{D}t} \tag{5}$$

where $\mathrm{D}(\ )/\mathrm{D}t$ is the material derivative. We assume that during the bubble-probe interaction, changes in the bubble velocity $\mathbf{u}_\mathrm{d}$ are larger than changes in the continuous-phase (water) velocity $\mathbf{u}_\mathrm{c}$, and happen faster than changes in the undisturbed flow ($t_\mathrm{b}/T \ll 1$, with t_b the bubble time scale and T the turbulent integral time scale); velocity changes due to bubble-probe interaction happen at a time scale $t_\mathrm{b} = (d + \Delta x)/u_\mathrm{d}$, while the turbulent integral time scale is proportional to $T \sim y/u_\mathrm{c}$. Thus, $\mathbf{F}_\mathrm{grad} \approx 0$ during the bubble-probe interaction as far as $(d+\Delta x)/u_\mathrm{d} \ll y/u_\mathrm{c}$, which holds within the bubbly flow region $C < 0.3$ for $y > (d+\Delta x)$. Another relevant assumption is that the intrusiveness of the probe does not lead to further local accelerations in the carrier flow.

The (quasi-)steady state drag force reads:

$$\mathbf{F}_\mathrm{d} = \frac{\pi d^2}{8}\rho_\mathrm{c} C_\mathrm{d}(\mathbf{u}_\mathrm{c} - \mathbf{u}_\mathrm{d})\,|\mathbf{u}_\mathrm{c} - \mathbf{u}_\mathrm{d}| \tag{6}$$

with C_d the drag coefficient. Extensive literature is available on the estimation of C_d for air bubbles in water [14; 15; 16]. Herein, we used the drag law for slightly contaminated systems [15]:

$$C_\mathrm{d} = \max\left[\min\left[\frac{24}{\mathrm{Re}_\mathrm{b}}\left(1 + 0.15\mathrm{Re}_\mathrm{b}^{0.687}\right), \frac{72}{\mathrm{Re}_\mathrm{b}}\right], \frac{8}{3}\frac{\mathrm{Eo}}{\mathrm{Eo}+4}\right] \tag{7}$$

where the Eötvös number is $\mathrm{Eo} = (g\,d^2\,(\rho_\mathrm{c} - \rho_\mathrm{d}))/\sigma$. The virtual mass force is related to the work needed to move the surrounding fluid when a bubble is accelerated. It can be written as [11]:

$$\mathbf{F}_\mathrm{vm} = \frac{\pi d^3}{6}\rho_\mathrm{c}\,C_\mathrm{vm}\left(\frac{\mathrm{D}\mathbf{u}_\mathrm{c}}{\mathrm{D}t} - \frac{\mathrm{d}\mathbf{u}_\mathrm{d}}{\mathrm{d}t}\right) \tag{8}$$

where C_vm is the virtual mass coefficient. Based on inviscid flow theory, $C_\mathrm{vm} \approx 0.5$ for spheres [11]. Different C_vm arise from bubble distortion such as in hyper-clean systems (Supplementary Note 4). Likewise after Supplementary Eq. (5), we assume that $|\ \mathrm{D}\mathbf{u}_\mathrm{c}/\mathrm{D}t\ |\ll|\ \mathrm{d}\mathbf{u}_\mathrm{d}/\mathrm{d}t\ |$ during the bubble-probe interaction and thus, $\mathrm{D}\mathbf{u}_\mathrm{c}/\mathrm{D}t \approx 0$.

The Basset history integral is the result of diffusion of vorticity from the bubble [14] and includes the effect of past accelerations, which are weighted non-linearly and inversely proportional to the elapsed time since the last acceleration, $\tau = t - s$, being s the time at which an acceleration happened. With increasing bubble Reynolds number (Re_b), the effect of this viscous memory force is reduced. Here, the expression of Mei et al. [17] is embraced as it accounts for finite Reynolds effects (that can be relevant as far as $\mathrm{Re}_\mathrm{b} > 0.1$):

$$\mathbf{F}_\mathrm{B} = 3\pi\rho_\mathrm{c}\nu_\mathrm{c} d\int_{-\infty}^{t}\mathcal{K}(\tau)\frac{\mathrm{d}(\mathbf{u}_\mathrm{c} - \mathbf{u}_\mathrm{d})}{\mathrm{d}s}\mathrm{d}s \tag{9}$$

Supplementary Table 1: Parameters of Supplementary Eq. (10) calibrated against data of Mei et al. [17] by minimizing the area difference between data and Supplementary Eq. (10) by adjusting N, while maintaining the maximum kernel value (M) and tail slope (α).

$\mathrm{Re_b}$	M	N	α
0.1	1.28	0.552	1.75
5	1.03	0.077	2.02
40	0.60	0.017	2.58
100	0.42	0.011	2.98

where $\mathcal{K}$ is the history force Kernel, representing the memory effect of the Basset force. We propose the following Kernel function, as shown in Supplementary Fig. 5, after performing a reanalysis of the numerical data of Mei et al. [17]:

$$\mathcal{K} = M\left(\frac{N}{N + \tau/\mathrm{Re_b}}\right)^{\alpha} \tag{10}$$

where M, N and α are empirical coefficients. The value of M corresponds to the value of $\mathcal{K}(\tau \rightarrow 0)$, whereas α is the power slope at large τ. Within the range of documented Reynolds numbers ($\mathrm{Re_b}$ = 0.1 to 100), empirical coefficients are linearly interpolated between values reported in Supplementary Table 1, whereas the bound values are taken outside this $\mathrm{Re_b}$ range.

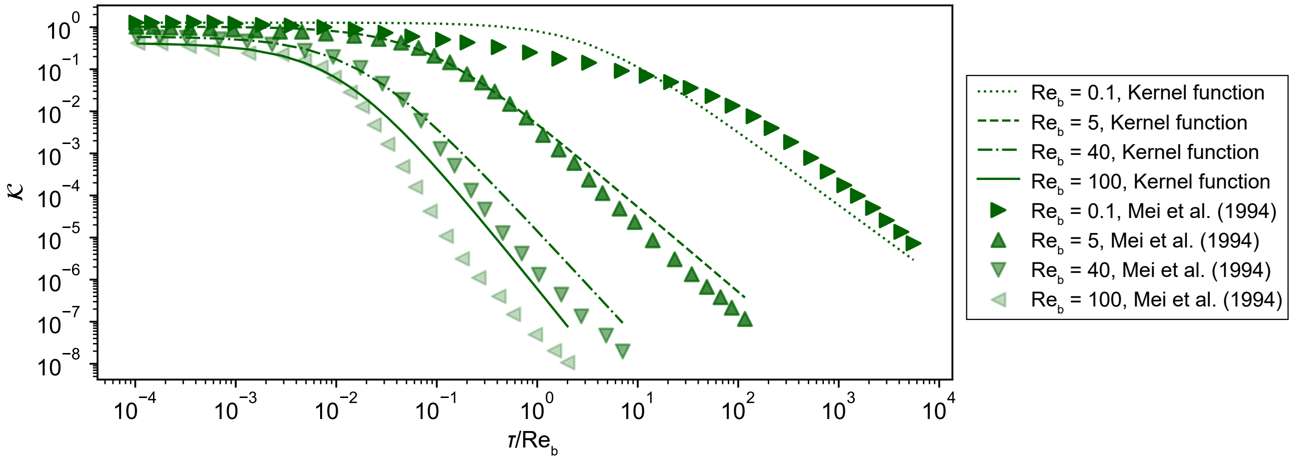

Supplementary Figure 5: Basset Kernel function. Lines for Supplementary Eq. (10) and markers for numerical simulation data of Mei et al. [17].

Bubble-probe interaction forces arise from the three-phase contact during bubble piercing [6] and from the impact of the bubble onto the tips [12]. The probe-wise component of the surface tension force can be expressed as [6]:

$$F_{\sigma} = -\beta\,\pi\,\sigma\,\Phi_{\mathrm{o}} \tag{11}$$

where σ is the surface tension coefficient, Φ_{o} is the outer needle diameter and $\beta \approx 1$ is a surface tension multiplier [6]. Bubble-probe interaction forces due to the impact of an air bubble comprise stagnation pressure against the probe (here we assume that kinetic energy acts as pressure over the probe tip area) and the surface deformation forces [following the observations of Lebanoff and Dickerson 12]:

$$F_{\mathrm{stag}} = -\frac{1}{8}\,C_{\mathrm{p}}\,\rho_{\mathrm{d}}\,u_{\mathrm{d}}^2\,\pi\,\Phi_{\mathrm{o}}^2 \tag{12}$$

$$F_{\mathrm{surf}} = -C_{\sigma}\sigma\,d\,\mathrm{We}^{1/4} = -C_{\sigma}\sigma\,d\left(\frac{\rho_{\mathrm{d}}u_{\mathrm{d}}^2 d}{\sigma}\right)^{1/4} \tag{13}$$

where C_{p} and C_{σ} are pressure and surface deformation coefficients, respectively. F_{stag} was found to be several orders of magnitude smaller for any flow situation (Supplementary Fig 6) and the pressure coefficient was simply taken as $C_{\mathrm{p}} = 1$. The rationale behind F_{surf} is that surface tension opposes bubble deformation when bubbles impact a probe needle, similar to droplets impacting needles [12]. The work of Lebanoff and Dickerson [12] suggests that impact and deformation forces experienced by a droplet impacting a transverse needle take place at a time scale proportional to $d/u_{\mathrm{d,corr}}$. Here, the impact and sliding of the bubble occurs along the leading needle and thus, these forces are considered continuously during the whole interaction ($\Delta x + d$). Furthermore, C_{σ} was calibrated based on available data sets for two different flow situations (Supplementary Note 4).

Combining Supplementary Eq. (3) with Supplementary Eqns. (4, 6, 8, 11, 12, 13) leads to the following force balance in probe-wise direction:

$$\underbrace{\frac{\pi d^3}{6}\rho_\mathrm{d}\frac{\mathrm{d}u_\mathrm{d}}{\mathrm{d}t}}_{\text{inertia}} = \underbrace{-\frac{\pi d^3}{6}(\rho_\mathrm{c}-\rho_\mathrm{d})\,g\cos\gamma}_{F_\mathrm{g}+F_\mathrm{p}} + \underbrace{\frac{\pi d^2}{8}\rho_\mathrm{c}C_\mathrm{d}\,(u_\mathrm{c}-u_\mathrm{d})\,|u_\mathrm{c}-u_\mathrm{d}|}_{F_\mathrm{d}} - \underbrace{\frac{\pi d^3}{6}\rho_\mathrm{c}\,C_\mathrm{vm}\left(\frac{\mathrm{d}u_\mathrm{d}}{\mathrm{d}t}\right)}_{F_\mathrm{vm}} + \underbrace{3\pi\rho_\mathrm{c}\nu_\mathrm{c}d\int_{-\infty}^{t}\mathcal{K}(\tau)\frac{\mathrm{d}(u_\mathrm{c}-u_\mathrm{d})}{\mathrm{d}s}\mathrm{d}s}_{F_\mathrm{B}}$$
$$-\underbrace{\beta\pi\sigma\Phi_\mathrm{o}}_{F_\sigma} - \underbrace{\frac{1}{8}C_\mathrm{p}\,\rho_\mathrm{d}\,u_\mathrm{d}^2\,\pi\,\Phi_\mathrm{o}^2}_{F_\mathrm{stag}} - \underbrace{C_\sigma\sigma\,d\left(\frac{\rho_\mathrm{d}u_\mathrm{d}^2 d}{\sigma}\right)^{1/4}}_{F_\mathrm{surf}} \quad (14)$$

where we have assumed that $F_\mathrm{grad} \approx 0$, $F_\mathrm{wall} \approx 0$, and $\mathrm{D}\mathbf{u}_\mathrm{c}/\mathrm{D}t \approx 0$. Note that the key assumptions for the solution of the force balance are listed in Supplementary Table 2 and that an order of magnitude analysis (Supplementary Fig. 6) has shown that F_B can be neglected for all practical flow situations.

Supplementary Table 2: Simplifications of the force balance

Equation	Force	Assumption	Comment	Supplementary Reference
Supplementary Equation 3	Wall forces	$\mathbf{F}_\mathrm{wall} \approx 0$	Lift and lubrication only relevant at $y < 1$ to $2\,d$	[13]
Supplementary Equation 5	Pressure gradient	$\mathrm{D}\mathbf{u}_\mathrm{c}/\mathrm{D}t \approx 0$	Flow gradients smaller than bubble deceleration, negligible probe intrusiveness	-
Supplementary Equation 9	Basset	$\mathbf{F}_\mathrm{B} \approx 0$	Basset force has negligible influence at high Re_b [17] (Supplementary Fig. 6)	[6]
Supplementary Equation 8	Virtual mass	$\mathrm{D}\mathbf{u}_\mathrm{c}/\mathrm{D}t \approx 0$	Flow gradients smaller than bubble deceleration, negligible probe intrusiveness	-

To better understand the relative contribution of each force component, we evaluated Supplementary Eq. 14 for bubbles of different diameters impacting a double-tip phase-detection probe (Δx = 4 mm, Φ_o = 0.6 mm) with undisturbed velocities of $u_\mathrm{d,corr}$ = u_c = 0.5 m s^{-1}, 3.5 m s^{-1}, 7.5 m s^{-1} and 12 m s^{-1}, where we assumed linear deceleration (Supplementary Fig. 4) between 5 % and 20 % along the probe. The results are shown in Supplementary Figs. 6a-d, indicating that Basset forces (F_B) and stagnation pressure forces (F_stag) – both not visible in Supplementary Fig. 6 – are considerably smaller than other force terms, regardless of the flow situation. Further, the bubble-probe interaction forces (F_σ, F_surf) exceed other forces at low velocities and small bubble diameters. For large velocities and diameters, the virtual mass force opposes greatly the deceleration, indicating that those bubbles experience a smaller interaction with the probe, reflecting results presented in Fig. 5.

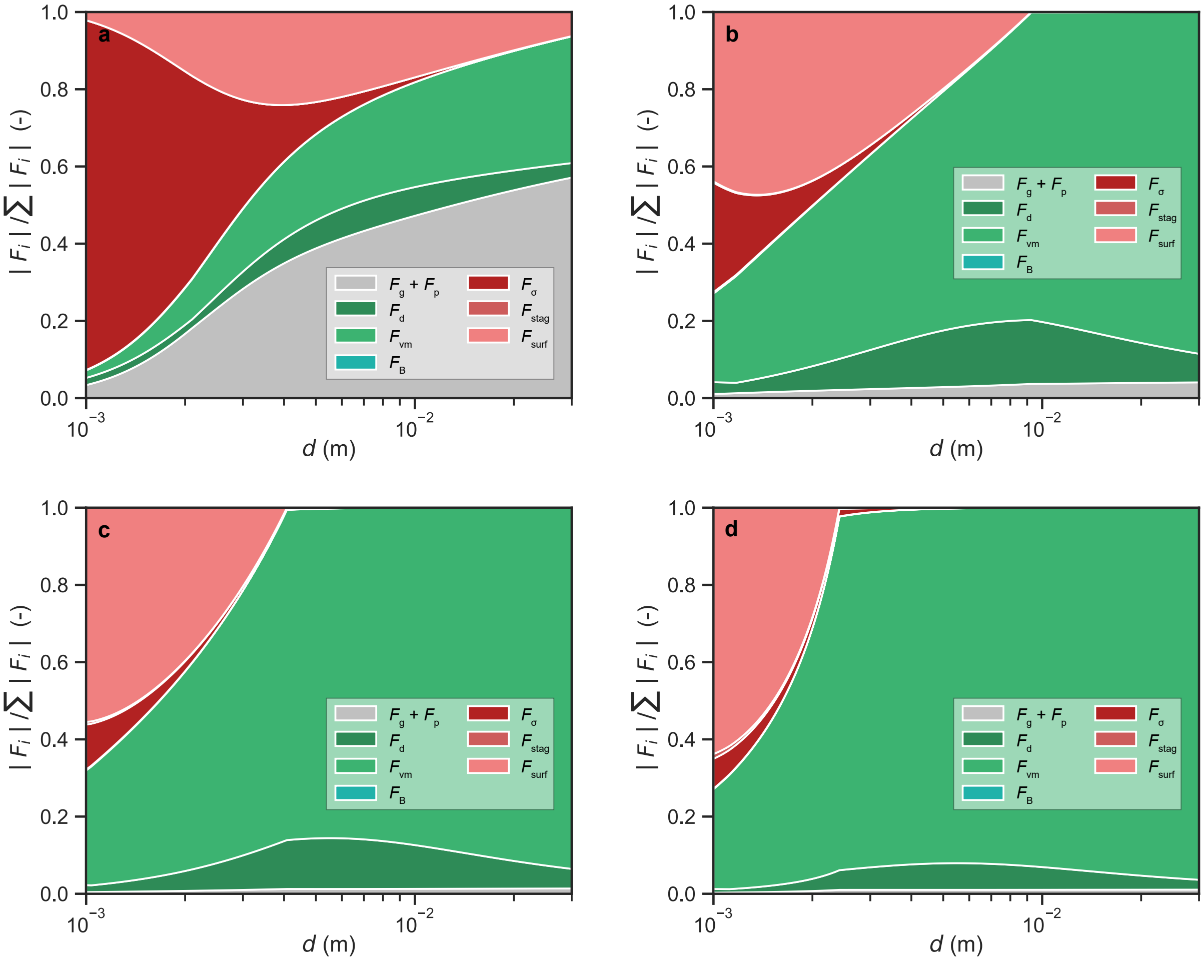

Supplementary Figure 6: Order of magnitude analysis for all forces of Supplementary Eq. (14) in the probe-wise direction at different impact velocities. Forces are expressed as relative, absolute (i.e, non-negative) terms, accounting for their maximum value during the deceleration process, i.e. gravity against the probe, drag and Basset forces considered after maximum deceleration for a linear deceleration and a probe geometry of Δx = 4 mm, Φ_o = 0.6 mm. **a** $u_{d,corr}$ = 0.5 m s^{-1}, assumed deceleration of 20%; **b** $u_{d,corr}$ = 3.5 m s^{-1}, assumed deceleration of 15%; **c** $u_{d,corr}$ = 7.5 m s^{-1}, assumed deceleration of 10%; **d** $u_{d,corr}$ = 12 m s^{-1}, assumed deceleration of 5%. Note that F_σ, F_{stag} and F_{surf} oppose to bubble movement, whereas F_d, F_{vm} and F_B push the bubble probe-wise against the needle if decelerated. $F_g + F_p$ can either act in one or another direction (or null) depending on the probe orientation.

Supplementary Note 4: Effects of semi-empirical coefficients and fluid properties on estimated velocities

Semi-empirical coefficients affect the result of the correction scheme. Relevant coefficients are summarised in Supplementary Table 3, comprising the drag coefficient (C_d), the virtual mass coefficient (C_{vm}), the surface tension multiplier (β), the stagnation pressure coefficient (C_p), the surface deformation coefficient (C_σ) as well as fluid properties. These coefficients may vary due to the flow conditions (e.g. Reynolds numbers), fluid properties (e.g. water quality, surfactants, particulate matter) or external effects such as temperature. Compiling data from literature, Supplementary Fig. 7 shows the effects of water quality on the rise velocity in water of different contamination levels (Supplementary Fig. 7a) as well as the effect of water quality on the virtual mass coefficients (Supplementary Fig. 7b). While distinct effects of the water quality can be found, the best-matching coefficients can be selected if the correct water quality conditions are used. Considering the data scatter in Supplementary Fig. 7a, b, we identified probability distributions for C_d, C_{vm},β and C_p (see second column in Supplementary Table 3)

Information on the effects of flow conditions on the deformation coefficient C_σ is missing in literature. Using the high-velocity data from the present study and assuming no slip, the coefficient C_σ was calibrated by matching the dispersed (CP) and continuous (LDA) phase velocities, solving $C_\sigma = \arg\min\left(U_d - \sum_{i=1}^{n} u_{d,corr}^{(i)} w^{(i)} / \sum_{i=1}^{n} w^{(i)}\right)^2$. The resulting values of C_σ are shown in Supplementary Fig. 7c as a function of the bubble Reynolds number Re_b. Available data from Verjrazka et al. [6] were also added as representatives for low Reynolds number flows. The values of C_σ increased from 0 for small Re_b to $\approx$ 13 for $\mathrm{Re}_b \approx 2 \cdot 10^4$ due to bubble deformation and lobe formation. For $\mathrm{Re}_b \gtrsim 2 \cdot 10^4$, the deformation coefficient C_σ decreased rapidly down to 0. This distinct trend was observed for all present data (Fig. 7c) and more research is needed to better identify if this sudden drop is linked to the break-up of bubbles or to larger bubble diameters, associated with less deformation under high-Re_b conditions. The overall trend of the data was best approximated by (Supplementary Fig. 7c):

$$C_\sigma = \max\left(0; -7.2 \cdot 10^{-8}\,\mathrm{Re}_b^2 + 0.002\,\mathrm{Re}_b - 0.59\right) \tag{15}$$

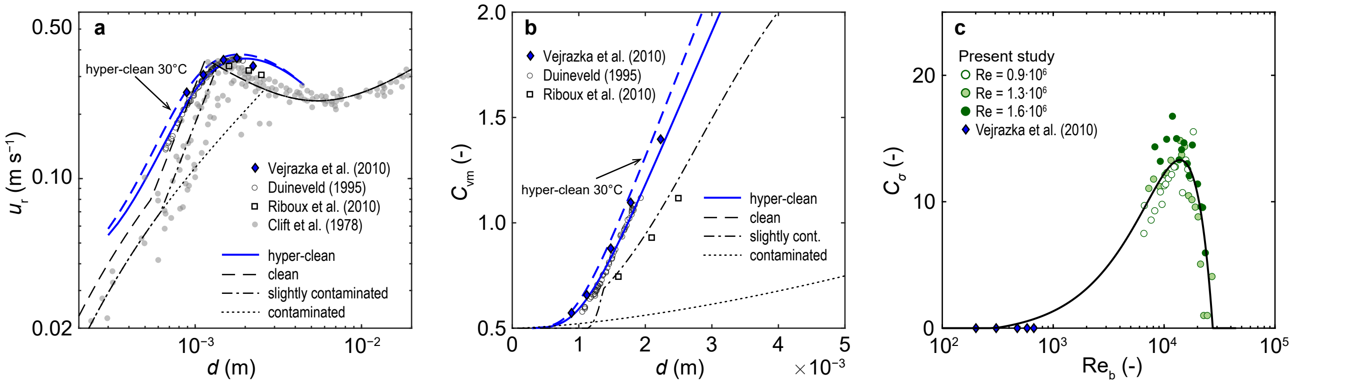

Supplementary Figure 7: Semi-empirical coefficients of the bubble force balance; **a** Bubble rise velocity calculations for hyper-clean [7] (with aspect ratio E after [8]), clean, slightly and fully contaminated systems [15]. **b** Virtual mass coefficient after [9] using excentricity estimated after [8] in hyper-clean, [18] slightly contaminated and [19] contaminated systems. **c** C_σ obtained from calibration, solid black line indicates Supplementary Eq. (15).

A sensitivity analysis was performed to identify the influence of semi-empirical parameter uncertainty and fluid properties on the developed correction scheme. This analysis was based upon a Monte Carlo simulation with 10,000 draws from assumed probability distributions (Supplementary Table 3) for flow conditions described in Supplementary Table 4. For each draw, the initial condition of the force balance $u_{d,corr} = u_c - u_r$ was calculated for a fixed u_c and then propagated to $u_{d,1,trail}$, i.e., the bubble velocity at the end of the interaction of the first interface with leading tip. Finally, the simulated measured bubble velocity was calculated as $u_{d,meas} = (u_{d,corr} + u_{d,1,trail})/2$.

Supplementary Figure 8 shows the resulting distributions of $u_{d,meas}$ around their mean values. For conditions with $u_{d,corr} \gtrsim 1$ m s^{-1}, the distributions were close to symmetrical and fairly narrow with 95% confidence bounds below ±5 to 10% (Supplementary Figs. 8a, b, d). However, for small velocities $u_{d,corr} < 1$ m s^{-1}, the confidence bound increased to ±20% (Supplementary Figs. 8c, e), which may be in the order of the bubble-probe interaction bias. In these cases, the water quality during the experiments (e.g. presence of surfactants, particulate matter, temperature) needs to be controlled more carefully to reduce the parameter uncertainty below the conservative assumptions made in Supplementary Table 3.

Supplementary Table 3: Semi-empirical coefficients and fluid properties used in the solution of the force balance for a slightly contaminated system (re-used tap water)

Parameter	Assumed distribution	Supplementary Reference
Drag coeff. C_d	Normal, $\mu_\mathrm{N} = f(\mathrm{Re_b}, \mathrm{Eo}), \sigma_\mathrm{N} = 0.2\,\mu_\mathrm{N}$	drag law [15], Supplementary Eq. (7)
Virtual mass coeff. C_vm	Triangular, $a = 0.4, b = 0.5, c = 1$	[9; 19; 18], Supplementary Fig. 7b
Surface tension multiplier β	Normal, $\mu_\mathrm{N} = 1, \sigma_\mathrm{N} = 0.1$	$\mu_\mathrm{N} \approx 1$ after [6]
Impact pressure coeff. C_p	Normal, $\mu_\mathrm{N} = 1, \sigma_\mathrm{N} = 0.1$	$\mu_\mathrm{N} \approx 1$ after [12]
Surface deformation coeff. C_σ	Triangular, $a = 0.5\,b$, $b = f(\mathrm{Re_b})$ (Supplementary Eq. 15), $c = 1.25\,b$	Supplementary Fig. 7c
water density ρ_c	Normal, $\mu_\mathrm{N} = f(\text{Temp., salinity, ...}), \sigma_\mathrm{N} = 0.002\,\mu_\mathrm{N}$	-
surface tension coeff. σ	Triangular, $a = 0.02$, $b = 0.073$, $c = 0.08$	[20]

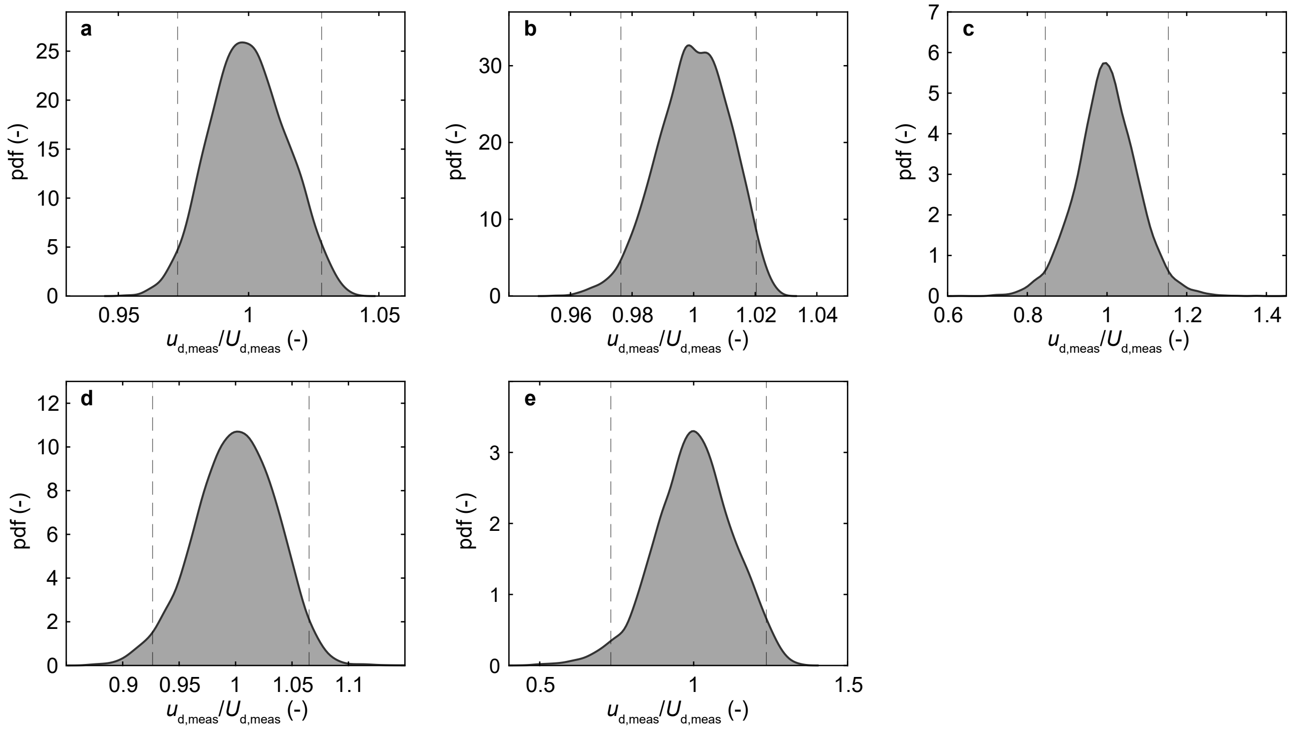

Supplementary Figure 8: Kernel density plot of the resulting velocity distributions due to uncertainty in coefficients of force balance (Supplementary Table 3) determined from a Monte Carlo simulation with 10,000 draws for flow conditions in Supplementary Table 4. Vertical dashed lines denote 95% confidence interval.

Supplementary Table 4: Flow parameters for Monte Carlo simulation in Supplementary Fig. 8.

Supplementary Fig. 8	γ (°)	d (mm)	$u_\mathrm{d,corr}$	$U_\mathrm{d,meas}/U_\mathrm{d,corr}$
**a**	90	3.0	1.0	0.94
**b**	90	1.0	5.0	0.96
**c**	180	3.5	0.24	0.85
**d**	180	1.0	3.2	0.90
**e**	0	4.0	0.21	0.95

Supplementary References

[1] Velte, C. M., George, W. K. & Buchhave, P. Estimation of burst-mode LDA power spectra. *Experiments in Fluids* **55**, 1674 (2014).

[2] Kramer, M., Hohermuth, B., Valero, D. & Felder, S. Best practices for velocity estimations in highly aerated flows with dual-tip phase-detection probes. *International Journal of Multiphase Flow* **126**, 103228 (2020).

[3] Auel, C., Albayrak, I. & Boes, R. M. Turbulence characteristics in supercritical open channel flows: Effects of Froude number and aspect ratio. *Journal of Hydraulic Engineering* **140**, 04014004 (2014).

[4] Nezu, I. & Nakagawa, H. Turbulence in open-channel flows. In *IAHR Monograph* (Balkema, Rotterdam, the Netherlands, 1993).

[5] Kramer, M., Valero, D., Chanson, H. & Bung, D. B. Towards reliable turbulence estimations with phase-detection probes: an adaptive window cross-correlation technique. *Experiments in Fluids* **60** (2019).

[6] Vejražka, J. *et al.* Measurement accuracy of a mono-fiber optical probe in a bubbly flow. *International Journal of Multiphase Flow* **36**, 533–548 (2010).

[7] Moore, D. W. The velocity of rise of distorted gas bubbles in a liquid of small viscosity. *Journal of Fluid Mechanics* **23**, 749–766 (1965).

[8] Aoyama, S., Hayashi, K., Hosokawa, S. & Tomiyama, A. Shapes of ellipsoidal bubbles in infinite stagnant liquids. *International Journal of Multiphase Flow* **79**, 23 – 30 (2016).

[9] Lamb, H. *Hydrodynamics, sixth edition* (Dover Publication, New York, 1932).

[10] Balachandar, S. & Eaton, J. K. Turbulent Dispersed Multiphase Flow. *Annual Review of Fluid Mechanics* **42**, 111–133 (2010).

[11] Crowe, C. T., Schwarzkopf, J. D., Sommerfeld, M. & Tsuji, Y. *Multiphase Flows with Droplets and Particles* (CRC Press, 2012), 2 edn.

[12] Lebanoff, A. P. & Dickerson, A. K. Drop impact onto pine needle fibers with non-circular cross section. *Physics of Fluids* **32**, 092113 (2020).

[13] Zaruba, A., Lucas, D., Prasser, H.-M. & Höhne, T. Bubble-wall interactions in a vertical gas–liquid flow: Bouncing, sliding and bubble deformations. *Chemical Engineering Science* **62**, 1591–1605 (2007).

[14] Clift, R., Grace, J. R. & Weber, M. E. *Bubbles, drops, and particles* (Dover Publications, 1978).

[15] Tomiyama, A., Kataoka, I., Fukuda, T. & Sakaguchi, T. Drag coefficients of bubbles: 2nd report, drag coefficient for a swarm of bubbles and its applicability to transient flow. *Transactions of the Japan Society of Mechanical Engineers Series B* **61**, 2810–2817 (1995).

[16] Tomiyama, A. Struggle with computational bubble dynamics. *Multiphase Science and Technology* **10**, 369–405 (1998).

[17] Mei, R., Klausner, J. F. & Lawrence, C. J. A note on the history force on a spherical bubble at finite reynolds number. *Physics of fluids* **6**, 418–420 (1994).

[18] Vakhrushev, I. & Efremov, G. Interpolation formula for computing the velocities of single gas bubbles in liquids. *Chemistry and Technology of Fuels and Oils* **6**, 376–379 (1970).

[19] Wellek, R. M., Agrawal, A. K. & Skelland, A. H. P. Shape of liquid drops moving in liquid media. *AIChE Journal* **12**, 854–862 (1966).

[20] Myint, W., Hosokawa, S. & Tomiyama, A. Terminal velocity of single drops in stagnant liquids. *Journal of Fluid Science and Technology* **1**, 72–81 (2006).
