# Supplementary material for: Velocity bias in intrusive gas-liquid flow measurements
Source: Nat Commun. 2021 Jul 5;12:4123. doi: 10.1038/s41467-021-24231-4 (PMC8257743; doi:10.1038/s41467-021-24231-4)
Supplement: Supplementary file 3 — Description of Additional Supplementary Files [file 41467_2021_24231_MOESM3_ESM.docx]

**Description of Additional Supplementary Files**

File name: Supplementary Data 1

Description: This file contains all source data from the Figures in the main text and Supporting Information. The data format is an Excel .xlsx container. Each sheet contains the data of one Figure as per the sheet name. Each sheet is divided to contain different subplots (if applicable) of the corresponding figure. The nomenclature is consistent with the main text and Supporting Information. Full references can be found in the main text and Supporting Information.
